# Reconstruction after wide excision of the nail apparatus for in situ or minimally invasive subungual melanoma: A retrospective case series

**DOI:** 10.1016/j.jpra.2025.10.027

**Published:** 2025-10-23

**Authors:** Luc Chouquet, Feriel Boukari, Thierry Balaguer, Henri Montaudié, Olivier Camuzard, Elise Lupon

**Affiliations:** a Department of Plastic and Reconstructive surgery, Institut Universitaire Locomoteur et du Sport, Pasteur 2 Hospital, 30 Avenue de la voie romaine, 06000, Nice, France; b Dermatology department, L’Archet Hospital, University Côte d’Azur, 151 Route de Saint-Antoine, 06200 Nice, France; c Université Côte d'Azur, CNRS, LP2M, 28 Avenue de Valombrose, 06100 Nice, France

**Keywords:** Subungual melanoma, Nail reconstruction, Wide local excision, Nail apparatus, Flap, Microsurgery, Oncology

## Abstract

**Introduction:**

Historically, subungual melanoma (SUM) was treated by amputation of the affected digit. Wide local excision (WLE) of the nail apparatus has since become a conservative alternative for in situ or minimally invasive lesions. While several reconstructive techniques have been described after WLE, few centers have reported their outcomes objectively. This study presents our series of reconstructions following WLE of the nail apparatus.

**Methods:**

We conducted a retrospective study at a university hospital, including patients referred by dermatologists for WLE and nail apparatus reconstruction between 2021 and 2024. Clinical, surgical, functional, and aesthetic outcomes were evaluated using validated scores (QuickDASH, Modified Mayo Wrist Score, AOFAS).

**Results:**

Ten patients were included. Reconstructions were performed with full-thickness skin grafts (*n* = 6), with or without dermal matrix (*n* = 4), and local flaps (*n* = 4). Five patients underwent immediate definitive reconstruction. Two recurrences occurred: one requiring dermal matrix removal, and the other necessitated amputation forinvasive SUM. Functional and aesthetic outcomes were satisfactory, with a mean follow-up of 19 months and no local recurrence in the remaining patients. The mean QuickDASH score was 25.5 ± 16.4 (range: 2.3–41). For the three foot cases, the mean AOFAS score was 86 ± 4.1 (range: 80–90).

**Conclusion:**

Nail apparatus reconstruction is feasible for in situ or minimally invasive SUM, particularly when the Breslow thickness is ≤0.5 mm. In invasive cases, immediate reconstruction risks being performed over residual tumor, supporting a two-stage approach, especially when donor site morbidity is expected. Techniques such as full-thickness skin grafts combined with a dermal matrix provide reliable functional and aesthetic outcomes. A two-stage approach is particularly valuable when oncologic margins are uncertain, as it reduces the risk of reconstructing over residual disease while preserving options with lower donor-site morbidity.

## Introduction

Melanoma is a malignant tumor arising from melanocytes and represents one of the most aggressive forms of skin cancer.^1^ Among the various subtypes, subungual melanoma (SUM) is a rare and often misdiagnosed form, accounting for approximately 1–3 % of all cutaneous melanomas.^2^ It typically involves the nail matrix and can be mistaken for benign conditions, leading to diagnostic and therapeutic delays.^3^

Historically, management of subungual melanoma frequently relied on digital amputation, based on the assumption of a high local recurrence risk and limited reconstructive options.^2^^,^^3^ However, recent advances have challenged this paradigm.^4^ Current recommendations favor function-preserving wide local excision (WLE) with oncologically sound margins, followed by immediate reconstruction. Although these approaches aim to maintain both oncologic safety and digital function, subungual melanoma remains a rare entity, and there is a paucity of data regarding optimal reconstructive strategies following nail apparatus excision.^1^^,^^4^

Beyond oncologic control, the management of SUM must also address the significant functional and aesthetic consequences of digital amputation.^5^^,^^6^ In recent years, there has been growing interest in WLE as a function-preserving alternative, although its indications, oncologic safety, and optimal reconstructive approach remain subjects of debate.^7^, ^8^, ^9^

In this retrospective monocentric case series, we describe our experience with reconstruction following wide excision of the nail apparatus for subungual melanoma, all performed by a single operator. We detail the surgical techniques used, analyze functional and aesthetic outcomes, and contribute to the limited body of literature addressing this challenging and uncommon condition.

## Materials and methods

### Study design

This is a single-center, single-surgeon retrospective case series study conducted between July 2024 and March 2025. It includes all patients who underwent reconstruction following WLE of the nail apparatus as part of the treatment for subungual melanoma.

This research project has been approved by the local institutional ethics committee (n IRB00014528_2025_14)

### Eligibility criteria

All patients referred by a dermatologist who underwent wide excision of the nail apparatus for subungual melanoma of the upper or lower limbs between January 2021 and October 2024 at our center were included.

Patients treated for non-melanoma nail tumors, those who declined participation, and those with insufficient follow-up, preventing adequate data collection, were excluded.

### Data collection

The following variables were collected for each patient:

- Demographic data: age, sex, occupation, and laterality (for upper limb cases).
- Comorbidities: history of diabetes, smoking, major comorbid conditions.
- Tumor characteristics: Breslow index, sentinel lymph node status.
- Surgical data: excision margins, reconstruction strategy (number of stages, number of steps, technique used, operative time).
- Postoperative follow-up: occurrence of major complications (infection, hematoma, flap failure) and minor complications (unplanned early consultation, wound dehiscence, delayed healing, return to the operating room for secondary revision).
- Follow-up duration.

Each patient was also asked to complete a functional assessment at least 6 months after the final surgical procedure:

- *At the upper limb*: QuickDASH (score 0–100) and Modified Mayo Wrist Score (score 0–100).
- *At the lower limb*: AOFAS score (American Orthopaedic Foot and Ankle Society; score 0–90).

## Results

### Wide local excision

**Figure 1:**
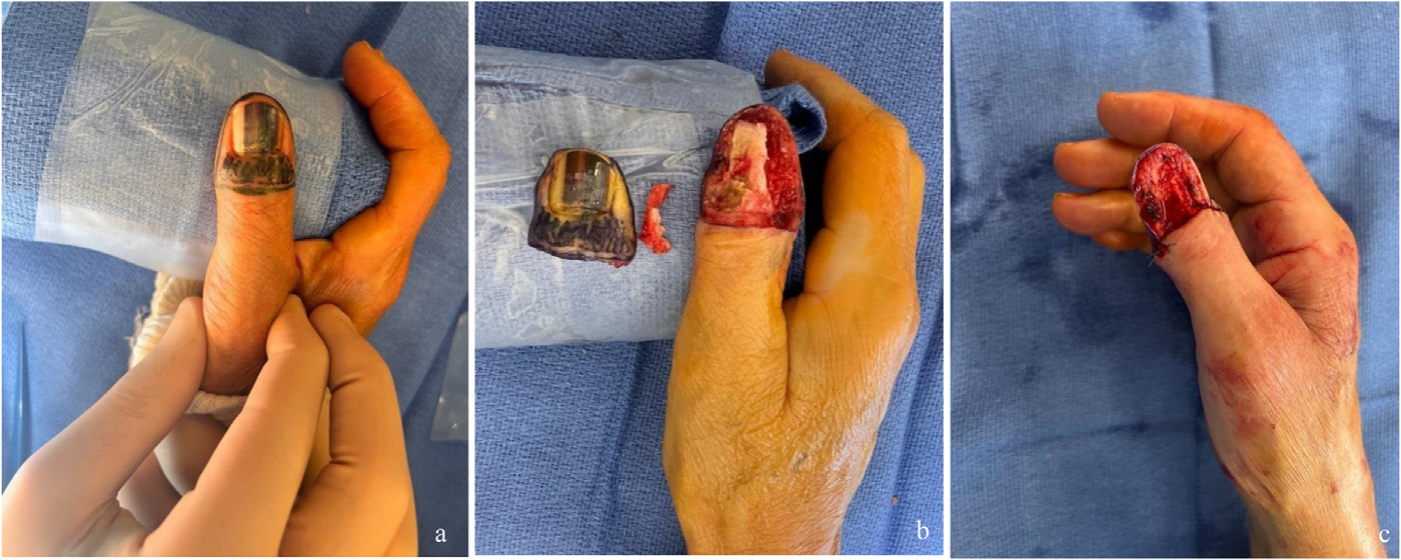
Photographs of the dorsal aspect of the thumb in a patient with SUM, treated with WLE. a. Intraoperative view before WLE. b, c. Intraoperative views after WLE, illustrating the extent of substance loss.

All patients included in our study underwent a biopsy prior to surgery. WLE was performed if the Breslow index was estimated to be equal or less than 0.5 mm. The surgical procedure was performed in the operating room under local anaesthesia and with a tourniquet applied to the root of the limb. The entire nail apparatus was excised, with peripheral margins ranging from 5 to 10 mm from the nail apparatus, depending on the patient and the prior biopsy. The deep margins corresponded to a subperiosteal dissection, in contact with the third phalanx of the ray (Figure 1).

### Population characteristics

A total of 10 patients were included, six of whom were women, with a mean age of 62.4 ± 10.8 years (range: 48–80). Seven patients were retired, and three were employed. There were no active smokers in the cohort. The main comorbidities included arterial hypertension in three patients, type two diabetes in one patient, and chronic obstructive pulmonary disease in one former smoker.

Six cases involved the upper limb—four affecting the thumb and two involving long fingers—while four cases involved the lower limb—three affecting the hallux and one the second toe.

### Per-operative data

**Figure 2:**
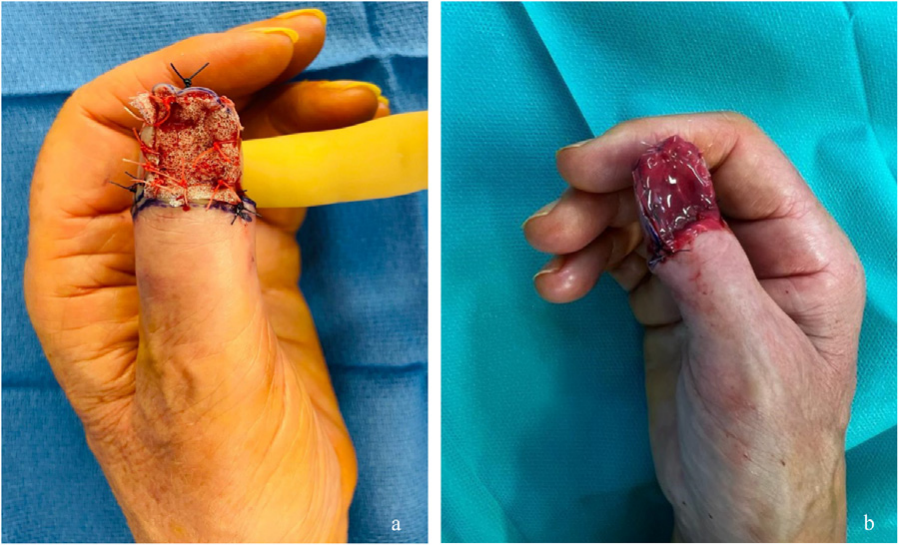
Photographs of the dorsal surface of the thumbs in patients with SUM, treated with WLE and reconstructed using a dermal matrix. a. Intraoperative view after WLE and placement of a fixed dermal matrix (Novosorb BTM). b. Intraoperative view after WLE and placement of a fixed dermal matrix (INTEGRA).

**Figure 3:**
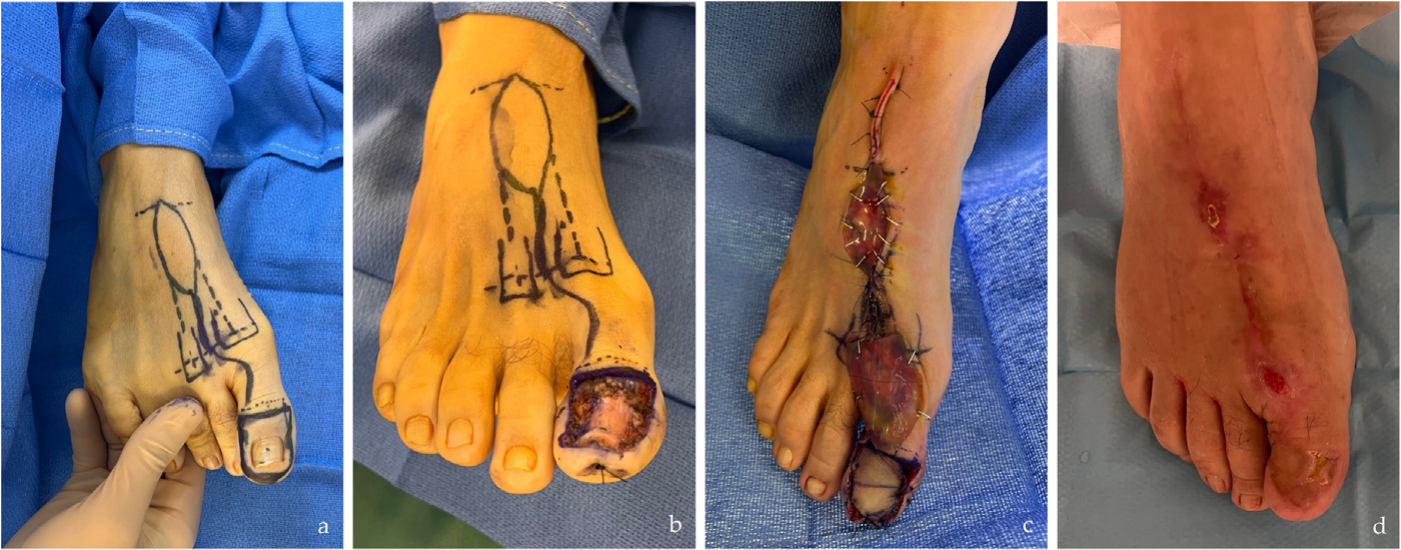
Photographs of the dorsal aspect of the right foot in a patient with hallux valgus treated with WLE and reconstructed using an intermetatarsal pedicle flap from the first space. a. Preoperative: marking of the flap on the donor site and identification of the location of the perforating artery of the dorsal metacarpal artery of the first space (FDMtA); b. Intraoperative: WLE completed, revealing loss of substance; c. Intraoperative: placement of the flap and its pedicle, application of INTEGRA dermal matrix to the donor site; d. Three months postoperatively: result with complete healing.

Five patients underwent single-stage surgery, and five patients underwent two-stage surgery (cases 1–5 in Table 1), with an average interval of 33 days between each procedure. The average duration of the first stage was 36 min, and that of the second stage was 27 min. Regarding upper limb cases, the most common reconstructive strategy was a two-stage approach with dermal matrix followed by a full-thickness skin graft, with a double-layer dermal matrix being placed at the same time in three out of six cases, and reconstruction being completed with a full-thickness skin graft taken from the inner side of the ipsilateral arm during the second stage (Figure 2). One of the patients underwent immediate, single-stage reconstruction using a subcutaneous adipofascial flap with a distal pedicle, and the two remaining patients with upper limb injuries underwent reconstruction using a full-thickness skin graft alone, although one of them had to undergo amputation of the affected limb later in their treatment. Regarding lower limb injuries, all reconstructions were performed in a single stage, using an intermetatarsal flap in two cases, a single-layer dermal matrix with a thin skin graft taken from the affected limb in one case, and direct closure with a pulp advancement flap in the case involving the second toe (Figure 3).

**Table 1:** Clinical characteristics and functional outcomes of 10 patients who underwent reconstruction after EEAU. total skin graft; TSG, thin skin graft; MMWS, Modified Mayo Wrist Score (score ranging from 100, no disability, to 0, maximum disability); Quick DASH, Quick Disability of the Arm, Shoulder and Hand score (score ranging from 0, no disability, to 100, maximum disability); (AOFAS, American Orthopaedic Foot and Ankle Score (score ranging from 0, maximum disability, to 90, no disability); NA, not applicable.

Case	Age	Dominant member	Sex	History	Location	Biopsy before WLE	Histopathology after WLE	Stage 1 surgery (Margins) (Duration)	Stage 2 surgery	Operation Interval	Side effects /complications	Score (%)	Cumu- lated Length of time off work	Follow-up Period
1	73	Right	M	HTN	Right thumb	Invasive SUM	Invasive SUM Breslow = 1,2mm	WLE + FTSG	Amputation trans P1 (17 min)	45 days	Brain metastasis, death	NA	None	24 months
2	68	Right	F	None	Right thumb	SUM in situ	SUM in situ	WLE + Dermal Matrix (29 min)	recovery of operating mar- gins + FTSG (33 min)	45 days	Hyposensitivity of the pinch, drop- ping objects Cold Hypersensitivity	MWS: 85 Quick DASH: 41	None	29 months
3	48	Right	F	None	Left Fg4	SUM in situ	SUM in situ	WLE (5 mm) (25 min)	FTSG (24 min)	15 days	Cold and Warm Hypersensitivity Tactical Hypersensitivity of dorsal fingertip	MWS: 80 Quick DASH :41	9 Weeks	18 months
4	66	Right	F	None	Right thumb	SUM in situ	Invasive SUM Breslow = 0.7mm	WLE (6 mm) + Dermal Matrix (29 min)	FTSG (33 min)	21 days	None	MWS :75 Quick DASH: 18	None	19 months
5	54	Right	M	None	Right thumb	SUM in situ	SUM in situ	WLE + Dermal Matrix (35 min)	FTSG (28 min)	21 days	None	NA	NA	13 months
6	80	Right	M	HTN, COPD	Right Fg2	SUM in situ	SUM in situ	WLE + Fascio-adipous flap (41 min)	None	None	Cold hyper- sensitivity Tactical hypersensitivity of dorsal fingertip	MWS :75 Quick Dash: 2.3	None	8 months
7	74	NA	F	HTN	Left big toe	SUM in situ	SUM in situ	WLE + Inter meta- tarsal flap (68 min)	None	None	None	AOFAS: 90	None	25 months
8	49	NA	F	Diabetic	Right big toe	Invasive SUM	Invasive SUM Bres- low = 0.8mm	WLE + Inter meta- tarsal flap (55 min)	None	None	None	AOFAS: 80	6 Weeks	24 months
9	60	NA	M	None	Right big toe	SUM in situ	SUM in situ	WLE + Dermal Matrix + STSG (39 min)	None	None	None	AOFAS: 90	None (stopped for per- sonal reasons)	13 months
10	52	NA	F	None	Left T2	SUM in situ	SUM in situ	WLE+ Pulp ad- vancement flap	None	None	Nail remnant	AOFAS: 87	1 day	7 months

### Postoperative data

Seven patients presented with melanoma in situ, and three had invasive melanoma, two of which were diagnosed preoperatively via biopsy. Among invasive melanomas, the mean Breslow thickness was 0.9 ± 0.21 mm (range: 0.7–1.2).

In two cases, the histological margins were positive after wide excision of the nail apparatus. These were managed either by amputation of the affected digit or by re-excision during the second stage of reconstruction, with no further oncologic treatment required.

The mean postoperative follow-up was 18 ± 7.1 months (range: 7–29). From an oncological standpoint, two patients had positive margins following WLE. One was managed by margin re-excision during the second stage of reconstruction, while the other underwent amputation of the affected digit within a month of the initial surgery and subsequently died from metastatic disease.

No major postoperative complications were observed, and no emergency reoperations were required. The most frequent complications were minor, mainly sensory-related, with some cases of delayed healing. One patient experienced hypoesthesia that impaired fine motor function, particularly when using forceps. Two patients reported pronounced contact hyperesthesia and sensitivity to temperature changes, including acrovascular phenomena, all in upper limb cases.

One patient (Case 10) developed a nail spicule at the surgical site, which caused discomfort while wearing shoes and required secondary excision. Graft hyperpigmentation was noted in only one case, following a two-stage reconstruction with a dermal substitute and subsequent full-thickness skin graft (FTSG).

Regarding functional and cosmetic outcomes, seven of the nine patients who underwent functional surgery completed the follow-up questionnaire after a mean delay of 19.2 months. This included four cases involving the fingers and three involving the toes.

**Figure 4:**
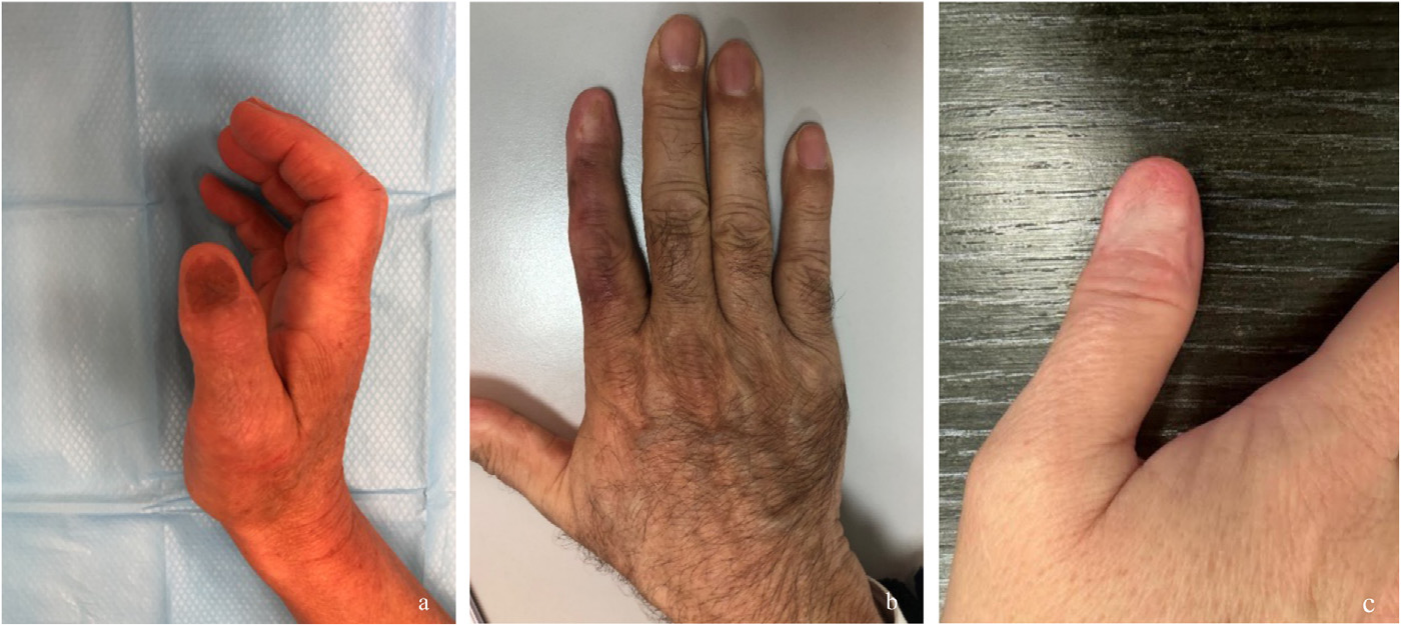
Photographs of the dorsal surface of digits treated with WLE and various reconstructive techniques. a. Right thumb reconstructed with a dermal matrix FTSG at 8-month follow-up, showing secondary hyperpigmentation of the graft. b. Right index finger reconstructed with a distal pedicled adipofascial flap at 8-month follow-up. c. Dorsal surface of a thumb in a patient with SUM treated with WLE and reconstructed with dermal matrix and FTSG at 24-month follow-up.

The mean QuickDASH score was 25.5 ± 16.4 (range: 2.3–41), where 0 indicates no disability and 100 indicates maximal disability. This included: two patients reconstructed with dermal matrix and FTSG (scores: 18 and 41); one patient with a distal pedicled adipofascial flap (score: 2.3); one patient with FTSG alone (score: 41) (Fig. 4).

The Modified Mayo Wrist Score, which ranges from 0 (maximum disability) to 100 (no disability), averaged 78.5 ± 4.8 (range: 75–85), with: Scores of 75 and 85 in the two patients reconstructed with dermal matrix and FTSG: 75 in the patient with a distal pedicled adipofascial flap and 80 in the patient with FTSG alone.

For the three cases involving the foot, the mean AOFAS score was 86 ± 4.1 (range: 80–90), where 90 indicates full function without disability.

A detailed breakdown of the results is presented in Table 1.

## Discussion

The demographic characteristics of our patients were consistent with the literature, with a slight predominance of females and a peak age between the 5th and 6th decades.^10^

In our series, all patients underwent a biopsy of the apparatus before WLE. As Chow et al. also mention^11^ routine preoperative biopsy allows us to confirm the diagnosis and, if not, to avoid mutilating and unnecessary surgery.

Excision with adequate margins (WLE) is now favored over amputation for cases of in situ or minimally invasive melanoma, although no consensus exists regarding a specific Breslow thickness threshold.^10^^,^^12^ In our practice, the dermatologist considers a Breslow depth of 0.5 mm as the limit for opting for wide local excision and reconstruction. This approach is supported by most studies, which report recurrence rates comparable to those observed with amputation.^2^^,^^7^^,^^13^, ^14^, ^15^

Given the proximity between the matrix, the nail bed, and the distal phalanx, we believe that a subperiosteal dissection is the minimum required, as leaving the periosteum in situ may carry a risk of residual matrix and subsequent spicule formation or local recurrence. In some cases, excision of a dorsal bone rod from the distal phalanx may be necessary when the biopsy reveals moderately advanced invasion. Some authors perform this procedure systematically, such as Moehrle et al., although the recurrence rate is comparable to that of subperiosteal dissections.^2^ As Sureda et al. point out, we believe that a minimum peripheral margin of 5 mm is essential both to prevent recurrence and to avoid residual matrix responsible for spicule formation.^16^

Although some studies have observed a higher rate of local recurrence after wide local excision (WLE),^17^ it should be noted that this technique is reserved for less aggressive tumors at an early stage of the disease. Furthermore, as pointed out by Kimyai-Asaidi et al.^18^ histopathological evaluation may be incomplete in such cases. This also explains the infrequent use of sentinel lymph node biopsy. On the other hand, amputation is generally performed for more advanced lesions with a higher metastatic potential. In the literature, a recurrence rate of between 15 % and 25 % is often observed in series, which corresponds to our series (two recurrences out of 10 cases). There is no established consensus on whether reconstruction should be performed immediately or delayed. In our practice, the more invasive the melanoma, the higher the risk of recurrence and incomplete resection, which leads us to favor guided healing or primary coverage with a dermal matrix, without morbidity at the donor site. Flaps and skin grafts are preferably reserved for the second stage of treatment of invasive melanomas or for the first and only stage of treatment of melanomas in situ.

There are numerous reconstruction options, which vary depending on the series.^1^^,^^19^, ^20^, ^21^, ^22^ The choice of technique depends on various factors such as the anatomical site, the functional importance of the finger and the priority given to structural strength or aesthetic outcome. Our series does not include any cases treated with free flaps, as we consider that the risk-benefit ratio is not favorable in terms of logistics, access to the operating theatre, donor site morbidity and outcome. We usually take our full-thickness skin grafts from the inner side of the arm on the same side, for accessibility (same surgical field), hairlessness, and the discretion of the scar from the donor site.^23^ We generally do not recommend secondary intention healing as a definitive reconstructive option because of the prolonged healing time.^5^, ^24^ When considered, it is more suitable for plantar locations, but in our practice its role remains limited for the upper limb.^8^

In our practice, we routinely use a dermal matrix after WLE in patients treated in two stages. These matrices serve to protect the bone of the distal phalanx, the healthy margin of the resection, and to provide a deep tissue framework, reducing the risk of hypersensitivity of a skin graft directly applied to the dorsal periosteum. For finger defects, we most often combine a dermal matrix with a full-thickness skin graft in a second stage, due to the functional and aesthetic advantages of these techniques. This combination ensures, on the one hand, a certain degree of dyschromia mimicking a preserved nail apparatus and, on the other hand, sensitivity through periosteal reinnervation of the graft, tempered by the interposition layer that is the matrix. The simplicity, reliability and effectiveness of this approach offer a favorable risk-benefit balance, which we usually prefer.

When it comes to covering the toes, pedicled flaps, such as the intermetatarsal flap we previously described, are often more suitable.^25^ Although they are more difficult to harvest than skin grafts, they ensure good trophicity and full skin thickness, allowing for a quick and lasting return to walking and wearing shoes. Their lack of sensitivity is desirable in this specific case so as not to restrict walking or wearing shoes. This is why we use the intermetatarsal flap, which gives good results, particularly in cases where the melanoma is in situ or slightly invasive and where single-stage reconstruction is indicated. In our experience, we did not encounter total flap loss, and technical refinements such as preoperative Doppler mapping (rather than relying solely on direct vision,^26^ together with preservation of a fatty cuff around the perforators, may facilitate safe harvest. The disadvantages include the presence of a dorsal scar along the toe and the interdigital commissure. In some cases, such as invasive melanomas, reconstruction using a skin graft combined with a dermal matrix may be a viable alternative.

**Figure 5:**
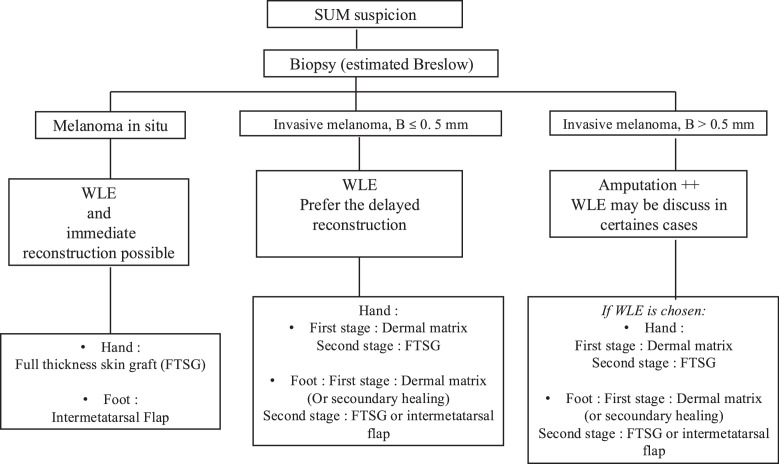
Proposed decision-making algorithm for the management of subungual melanoma, outlining indications for amputation versus wide local excision, and preferred reconstructive strategies according to Breslow thickness, tumor invasiveness, and anatomical site.

Based on our institutional experience and current literature,^8^^,^^27^ we have developed a practical decision-making algorithm to guide the choice between amputation and wide local excision, as well as the timing and type of reconstruction, according to tumor stage and anatomical location (Fig. 5).

This study has several limitations. First, it reports a small, single-center retrospective series, which limits the generalizability of our findings. Second, the cohort is heterogeneous in terms of reconstruction techniques, reflecting individualized decision-making rather than a standardized protocol. Third, the mean follow-up period was relatively short, which may underestimate the rate of late local recurrences or long-term functional sequelae. Finally, the proposed decision-making algorithm is largely based on expert opinion and institutional practice, supported by limited published data. Nevertheless, this study was conceived as a pilot series aimed at reporting the first outcomes of this reconstructive approach. With a mean follow-up of 18 months, our results already provide valuable insight into reconstructive feasibility and functional recovery. Importantly, very few studies have specifically addressed reconstruction in this clinical setting, which underscores the relevance of presenting these early findings.^8^ Because the primary objective of this work was to focus on reconstructive strategies rather than oncologic outcomes, the data reported here represent only an initial step. Future multicenter studies with larger sample sizes, standardized reconstructive strategies, and longer follow-up are needed to confirm these preliminary results.

## Conclusion

WLE is now an established option for treating subungual melanoma with a low Breslow thickness (≤0.5 mm). The main challenge is selecting the most appropriate reconstructive method for each location and patient profile. Full-thickness skin grafts and dermal matrices provide satisfactory functional and aesthetic outcomes for upper limb lesions, while the intermetatarsal flap is especially useful for lower limb reconstruction. To date, no consensus exists regarding the optimal number of reconstructive stages. A two-stage strategy is supported by oncologic safety, particularly in invasive melanomas, and by the need to minimize donor site morbidity associated with autologous reconstruction.

## Declaration of Competing Interest

The authors declare no conflicts of interest.
